# Early Developmental Characteristics and Features of a Three-Dimensional Retinal Organoid Model of X-Linked Juvenile Retinoschisis

**DOI:** 10.3390/ijms25158203

**Published:** 2024-07-27

**Authors:** Jung Woo Han, Hun Soo Chang, Sung Chul Park, Jin Young Yang, Ye Ji Kim, Jin Ha Kim, Hyo Song Park, Han Jeong, Junwon Lee, Chang Ki Yoon, Hyung Gon Yu, Se Joon Woo, Jungmook Lyu, Tae Kwann Park

**Affiliations:** 1 Department of Ophthalmology, Soonchunhyang University Bucheon Hospital, Soonchunhyang University College of Medicine, 170, Jomaru-ro, Bucheon 14584, Republic of Korea; 106236@schmc.ac.kr (J.W.H.); 138538@schmc.ac.kr (S.C.P.); 114733@schmc.ac.kr (J.H.K.); 124533@schmc.ac.kr (H.S.P.); 2 Department of Ophthalmology, Soonchunhyang University College of Medicine, Cheonan 31151, Republic of Korea; 3 Department of Microbiology, Soonchunhyang University College of Medicine, Cheonan 31151, Republic of Korea; intron@schmc.ac.kr; 4 Department of Interdisciplinary Program in Biomedical Science, Soonchunhyang Graduate School, Soonchunhyang University Bucheon Hospital, Bucheon 14584, Republic of Korea; yeiji77@naver.com; 5 Laboratory of Molecular Therapy for Retinal Degeneration, Soonchunhyang University Bucheon Hospital, Soonchunhyang University College of Medicine, Bucheon 14584, Republic of Korea; roswellgirl111@gmail.com; 6 Institute of Vision Research, Department of Ophthalmology, Severance Eye Hospital, Yonsei University College of Medicine, Seoul 03722, Republic of Korea; jnghan11@naver.com; 7 Brain Korea 21 Project for Medical Science, Yonsei University College of Medicine, Seoul 03722, Republic of Korea; 8 Institute of Vision Research, Department of Ophthalmology, Gangnam Severance Hospital, Yonsei University College of Medicine, Seoul 06273, Republic of Korea; bravewon@yuhs.ac; 9 Department of Ophthalmology, Seoul National University Hospital, Seoul National University College of Medicine, Seoul 03080, Republic of Korea; syst18@gmail.com; 10 Retina Center, The Sky Eye Institute, Seoul 06536, Republic of Korea; hgonyu@snu.ac.kr; 11 Department of Ophthalmology, Seoul National University Bundang Hospital, Seoul National University College of Medicine, Seongnam 13620, Republic of Korea; sejoon1@snu.ac.kr; 12 Department of Medical Science, Konyang University, Daejun 32992, Republic of Korea; lyujm@konyang.ac.kr

**Keywords:** induced pluripotent stem cells, organoids, retina, retinoschisis

## Abstract

X-linked juvenile retinoschisis (XLRS) is a hereditary retinal degeneration affecting young males caused by mutations in the retinoschisin (*RS1*) gene. We generated human induced pluripotent stem cells (hiPSCs) from XLRS patients and established three-dimensional retinal organoids (ROs) for disease investigation. This disease model recapitulates the characteristics of XLRS, exhibiting defects in RS1 protein production and photoreceptor cell development. XLRS ROs also revealed dysregulation of Na/K-ATPase due to RS1 deficiency and increased ERK signaling pathway activity. Transcriptomic analyses of XLRS ROs showed decreased expression of retinal cells, particularly photoreceptor cells. Furthermore, relevant recovery of the XLRS phenotype was observed when co-cultured with control ROs derived from healthy subject during the early stages of differentiation. In conclusion, our in vitro XLRS RO model presents a valuable tool for elucidating the pathophysiological mechanisms underlying XLRS, offering insights into disease progression. Additionally, this model serves as a robust platform for the development and optimization of targeted therapeutic strategies, potentially improving treatment outcomes for patients with XLRS.

## 1. Introduction

X-linked juvenile retinoschisis (XLRS) is a bilateral, inherited, progressive retinal degenerative disease observed in young males, caused by loss-of-function mutations in the retinoschisin (*RS1*) gene [1,2]. Clinical manifestations in affected individuals encompass varying degrees of progressive central vision loss, characterized by radial streaks originating from foveal schisis and the splitting of inner retinal layers in the peripheral retina [3]. Additionally, defects in signal transmission from photoreceptors to bipolar cells, as visualized by electroretinogram recordings, are observed [4,5]. These reveal a characteristic reduction in the b-wave amplitude, whereas the a-wave remains relatively unaffected. Predominantly affecting young males, XLRS has prevalence estimates ranging from 1 in 5000 to 1 in 20,000 [6,7,8].

*RS1*, the gene implicated in XLRS, encodes a highly conserved secreted extracellular protein known as retinoschisin. Comprising six exons, retinoschisin encodes a 224-amino-acid protein featuring a hydrophobic leader sequence with a consensus signal peptidase cleavage site, facilitating its export as oligomers [9,10]. The retinoschisin primarily localizes to the extracellular surfaces of the inner segments of both rod and cone photoreceptors in the retina, as well as in bipolar cells and two plexiform layers [11,12]. Playing a crucial role, retinoschisin is instrumental in maintaining the cellular organization of the retina. Studies using *RS1* knockout (KO) mice have demonstrated the essential nature of retinoschisin in preserving cell-to-cell interactions among retinal cells [13]. However, interspecies variations, including differences in retinal structure and life rhythm, impose limitations on the applicability of animal models in mechanistic and particularly preclinical studies. Furthermore, numerous mouse models of retinal disorders have fallen short in replicating disease-relevant phenotypes [14,15]. These observations raise concerns regarding the suitability of the mouse in vivo model as a reliable surrogate for investigating the function of retinoschisin in the retinal development of XLRS.

In recent years, the integration of patient-specific induced pluripotent stem cells (iPSCs) with advanced differentiation techniques has emerged as an invaluable cell source for disease modeling [16,17,18,19,20]. This approach enables the faithful reproduction of primary disease tissue characteristics following appropriate induction. Notably, iPSCs offer a platform for the exploration and functional validation of genotype–phenotype relationships within patient-specific genetic backgrounds. A significant milestone in this trajectory is the successful generation of retinal organoids (ROs) from iPSCs, representing a breakthrough in recapitulating retinogenesis [21,22]. ROs derived from pluripotent stem cells not only provide a physiologically relevant model but also facilitate detailed analysis through transcriptomic approaches [23,24,25]. This capability has led to the application of iPSCs in studying human hereditary retinopathies, leveraging their potential for disease modeling and mechanistic investigations [26].

In this study, we enrolled two XLRS patients with *RS1* mutations to generate patient-specific human iPSCs (hiPSCs). Subsequently, we established three-dimensional (3D) ROs from these patient-specific hiPSCs to model XLRS for disease investigation. Morphological analysis using immunohistochemistry, as well as transcriptomic analysis by total RNA sequencing, was performed to characterize early XLRS organoids and compared them to control organoids derived from healthy subject with no mutation in RS1 gene. Additionally, our results revealed a partial recovery of the XLRS phenotype when co-cultured with control ROs during the early stages of development.

## 2. Results

### 2.1. Generation of 3D ROs from XLRS Patient-Specific hiPSCs

**Figure 1:**
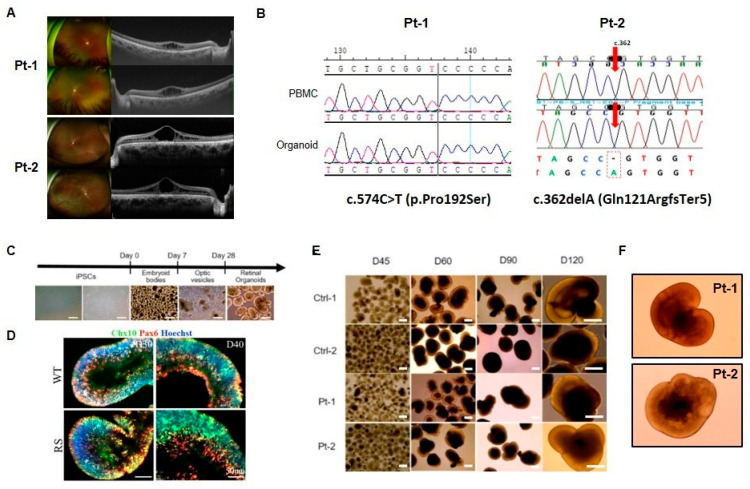
Generation of three-dimensional retinal organoids (ROs) from X-linked juvenile retinoschisis (XLRS) patient-derived human induced pluripotent stem cells (hiPSCs). (**A**) Color fundus photograph and optical coherence tomography images (right panel) of XLRS patient. (**B**) Sanger sequencing of retinoschisin (RS1) mutation in hiPSCs. Two patients carry *RS1* mutations at c.574C>T and c.365delA. The red arrows indicate the site of adenine deletion (**C**) Main steps of hiPSC-derived RO development in vitro: hiPSC colony, embryoid body formation, neuroretinal domain, and ROs (Scale bars: 200 µm) (**D**) Immunofluorescence staining of Chx10 (green) and Pax6 (red) in retinal progenitors at 30–40 days of differentiation (scale bar = 50 µm). (**E**) Morphology of ROs up to day 120 of differentiation in two controls (Ctrl-1 and Ctrl-2), Pt-1 and Pt-2 (Scale bars: 200 µm). (**F**) Splitting (schisis) regions of Pt-1 and Pt-2 ROs.

One control RO (Ctrl-1) was generated from the American Type Culture Collection (ATCC)-DYR0100 hiPSC, derived from a foreskin fibroblast cell line. One control donor (Ctrl-2) and two patients diagnosed with XLRS (Pt-1 and Pt-2) were enrolled in this study. Control-2 was confirmed to be healthy subject with no mutation in the *RS1* and other ocular disease related gene (Supplementary Table S1). Fundoscopic examination showed a spoke wheel pattern radiating from the fovea and a domelike elevation of a thin layer of retina (Figure 1A). Pt-1 and Pt-2 were found to carry *RS1* mutations respectively at c.574C>T (p.Pro192Ser) and c.365delA (Gln121ArgfsTer5). Peripheral blood mononuclear cells (PBMCs) from XLRS patients and control donor were reprogrammed into hiPSCs using the non-viral episomal plasmid protocol. Genotyping of the XLRS patient-derived hiPSC clones confirmed the presence of the respective *RS1* mutations at c.574C>T and c.365delA (Figure 1B). We generated 3D ROs (Figure 1C) using hiPSCs derived from normal iPSC and control donor (Ctrl-1 and Ctrl-2) and individuals with XLRS (Pt-1 and Pt-2). The hiPSC-derived aggregates were gradually formed from embryoid bodies (EBs). Optic vesicles were manually collected between days 25 and 28 and subsequently formed 3D ROs when cultured in suspension. After 28 days of differentiation, the ROs developed distinctive morphological and phenotypic characteristics, including a bright, stratified layer toward the periphery (Figure 1C). At 30 and 40 days of differentiation, both control and patient ROs exhibited widespread expression of the retinal progenitor marker CHX10 and PAX6, with no difference between control and patient ROs (Figure 1D). We then cultured and sustained control and patient ROs for up to 120 days (Figure 1E). The splitting of the retina between the outer and inner core layers was observed in patient ROs, but not in the majority of cases (Figure 1F).

### 2.2. Impaired Photoreceptor Cell Development in XLRS ROs

**Figure 2:**
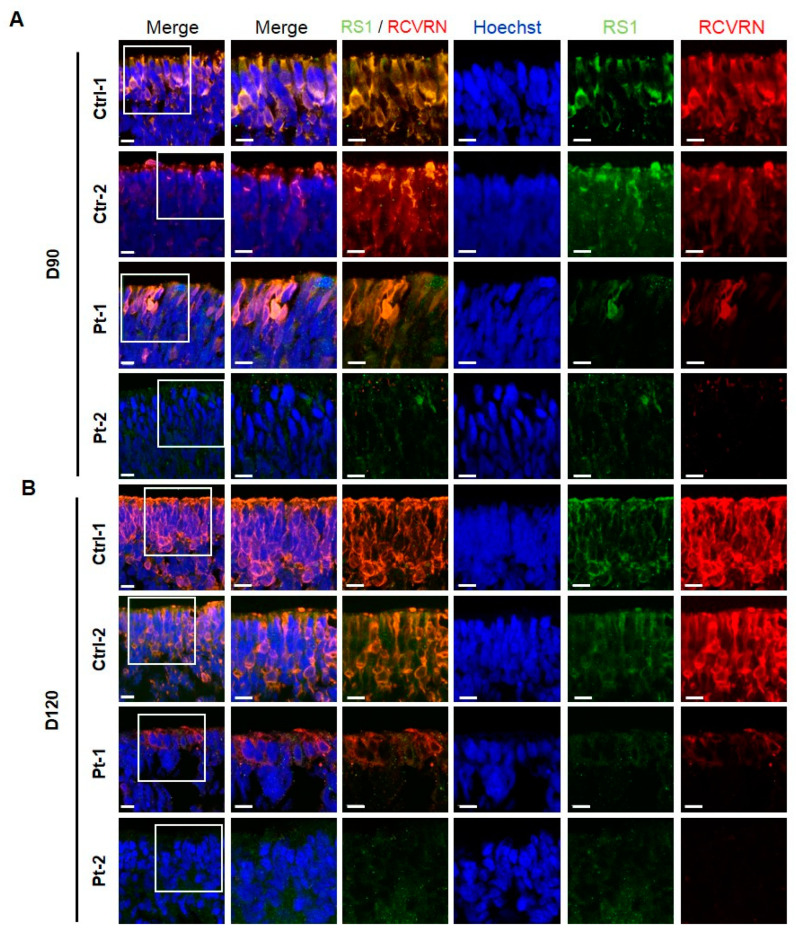
Representative images of immunofluorescence for retinoschisin (RS1) and photoreceptor cells in control and patient retinal organoids (ROs) at day 90 and 120 of differentiation. (**A**) Immunofluorescence staining of RS1 (green) and photoreceptor marker recoverin (RCVRN) (red) in the outer layer of control and patient ROs at days 90. (**B**) Immunofluorescence staining of RS1 (green) and photoreceptor marker RCVRN (red) in the outer layer of control and patient ROs at days 120 (scale bar = 50 µm).

We investigated the expression of RS1 in ROs using immunofluorescence staining, and to confirm any changes in developing photoreceptors. The expression of RS1 increased in the outer layer of developing ROs at 90 and 120 days, with a significant decrease observed in XLRS patient ROs compared to control ROs (Figure 2A,B). Similarly, the expression of recoverin (RCVRN) significantly increased in control ROs, whereas it was significantly decreased in patient ROs at 90 and 120 days (Figure 2A,B).

**Figure 3:**
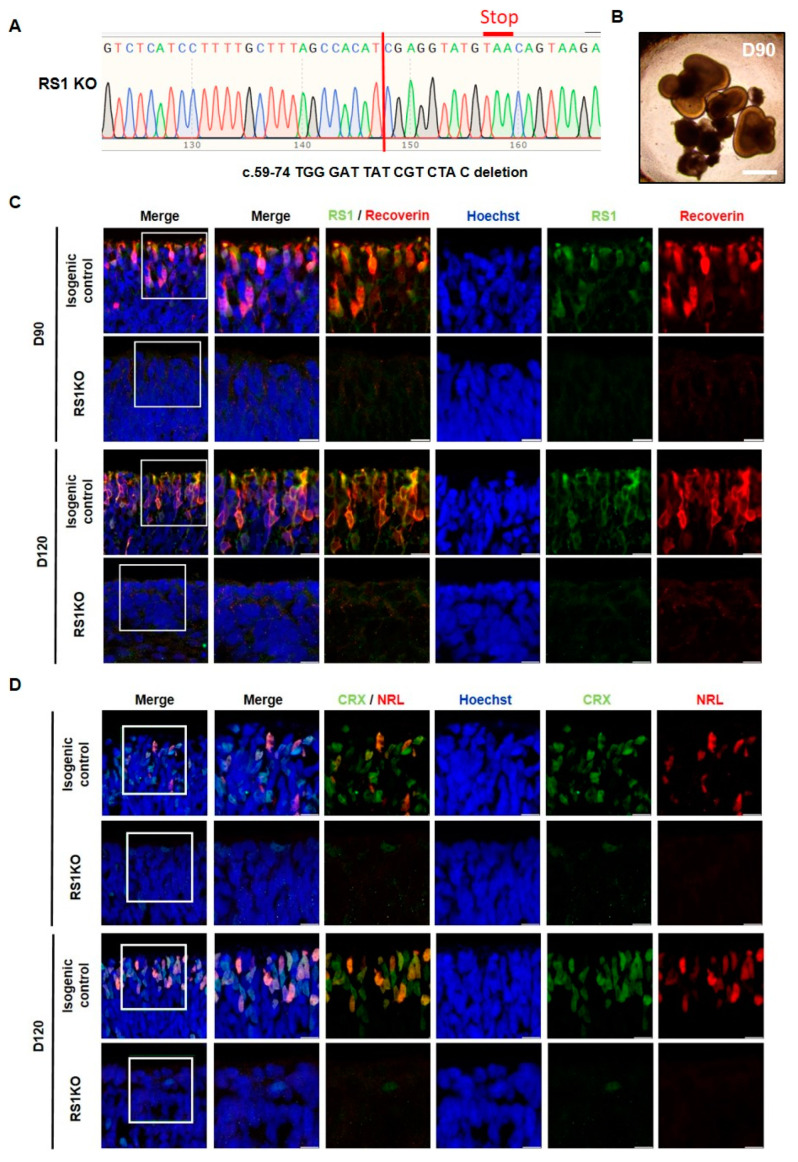
Characterization of retinal organoids (ROs) derived from clustered regularly interspaced short palindromic repeats (CRISPR)/associated protein 9 (Cas9)-mediated retinoschisin (RS1) mutated induced pluripotent stem cells (iPSCs). (**A**) Target sequence of CRISPR/Cas9 gene editing is located in exon 2 of the RS1 gene. Sanger sequencing of edited iPSC showed 15 base pair deletion (c.59–74, red line). (**B**) Bright-field images of retinoschisin knockout (RS1-KO) ROs at days 90 (Scale bars: 200 µm). (**C**) Immunofluorescence staining of RS1 (green) and photoreceptor marker recoverin (RCVRN) (red) in the outer layer of isogenic control and RS1-KO ROs at days 90 and 120 (scale bar = 50 µm). (**D**) Immunofluorescence staining of CRX (green) and NRL (red) in the outer layer of isogenic control and RS1-KO ROs at days 90 and 120 (scale bar = 50 µm).

To further ensure our findings, we employed CRISPR/Cas9 gene editing to generate RS1 KO iPSCs from a healthy donor iPSC line. We designed a single guide RNA (gRNA) target sequence and a homologous template to induce a stop codon via frameshift deletion mutation in Exon 2 of the *RS1* gene (Figure 3A). Subsequently, we differentiated the RS1-KO iPSCs into 3D ROs (Figure 3B). Consistently with previous data, RS1-KO ROs exhibited no expression of RS1 and RCVRN compared to isogenic control ROs at 90 and 120 days (Figure 3C). The expression of NRL and CRX was markedly decreased in RS1-KO ROs compared to isogenic control ROs at 90 and 120 days (Figure 3D). These results showed that photoreceptors do not develop in RS1-KO ROs.

### 2.3. Na/K-ATPase Dysregulation by RS1 Deficiency in XLRS ROs

**Figure 4:**
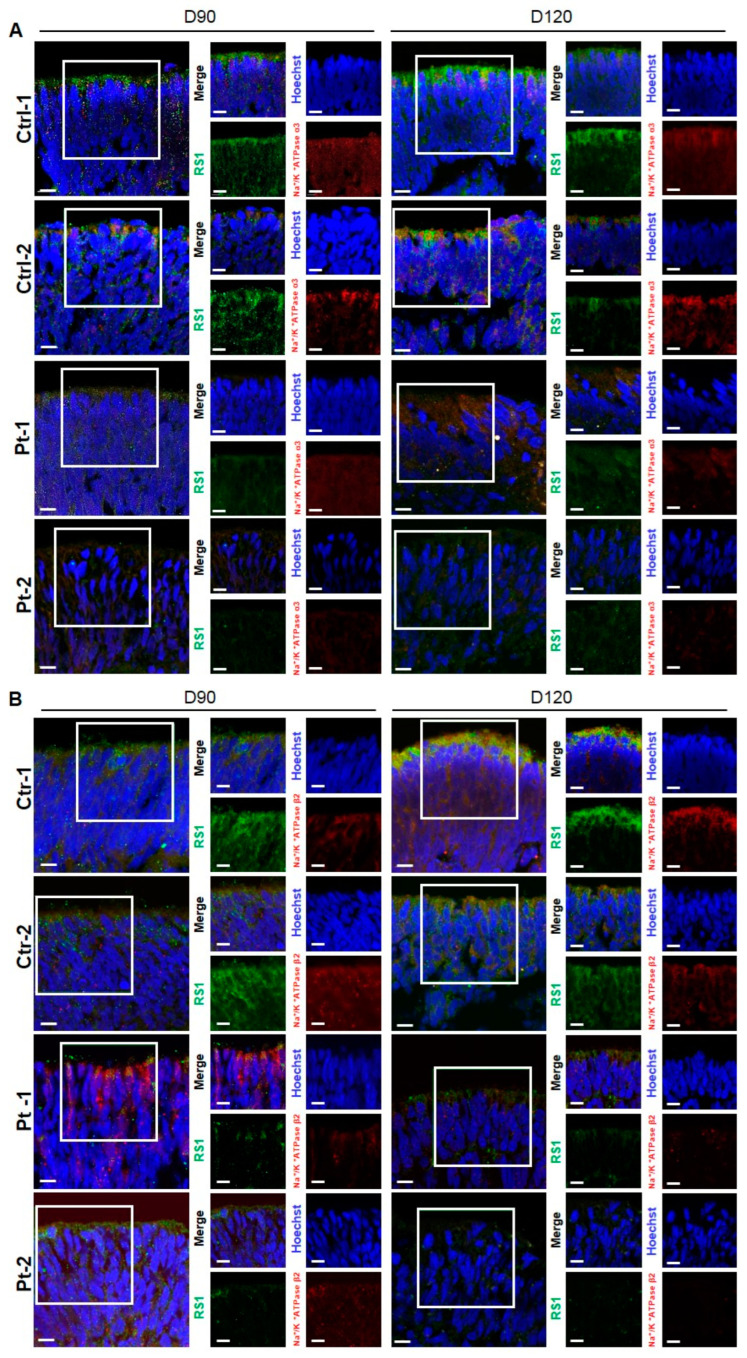
Representative images of immunofluorescence for retinoschisin (RS1) and Na/K-ATPase in control and patient retinal organoids (ROs) at day 90 and 120 of differentiation. (**A**) Immunofluorescence staining of RS1 (green) and retinal Na/K-ATPase subunits α3 (ATP1A3) (red) in the outer layer of in the outer layer of control and patient ROs at days 90 and 120 (scale bar = 50 µm). (**B**) Immunofluorescence staining of RS1 (green) and retinal Na/K-ATPase subunits β2 (ATP1B2) (red) in the outer layer of control and patient ROs at days 90 and 120 (scale bar = 50 µm).

The retinal Na/K-ATPase, comprised of subunits α3 (ATP1A3) and β2 (ATP1B2), plays a crucial role in anchoring RS1 to the plasma membrane [27]. In this study, we investigated the expression of Na/K-ATPase in conjunction with RS1 in ROs. As mentioned above, strong RS1 signals were observed in the outer layer of control ROs. Similarly, a comparable distribution was observed for ATP1A3 and ATP1B2. Focusing on the RS1-containing retinal layers, we identified colocalization between RS1 and the Na/K-ATPase subunits ATP1A3 and ATP1B2 at 90 and 120 days (Figure 4A,B). In XLRS ROs, both RS1 and ATP1A3 and ATP1B2 were observed to be significantly decreased compared with control ROs (Figure 4A,B).

### 2.4. Increased ERK Signaling in XLRS ROs

**Figure 5:**
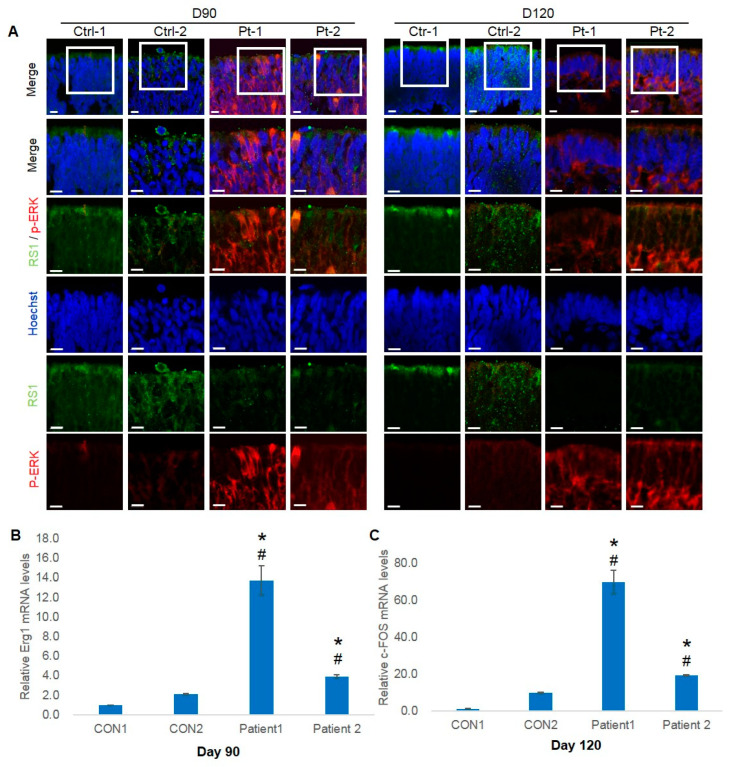
Influence of retinoschisin (RS1) on the extracellular signal-regulated kinase (ERK) pathway in control and patient retinal organoids (ROs). (**A**) Immunofluorescence staining of RS1 (green) and p-44/42 (red) in control and patient ROs at days 90 and 120 (scale bar = 50 µm). (**B**,**C**) FOS and EGR1 expression in control and patient ROs at days 90 and 120. *n* = 3 per group; * *p* < 0.05, between Ctrl-1 and Pt-1; # *p* < 0.05, between Ctrl-2 and Pt-2, by one-way analysis of variance.

A previous study investigated the upregulation of mitogen-activated protein (MAP) kinase activity during early retinal development in RS1 KO mice [28]. To corroborate these findings, we assessed the phosphorylation of Erk1/2, as well as EGR1 and FOS, prominent target genes of activated MAP kinases, in both Control and XLRS ROs. The expression of p-Erk1/2 in patient ROs was markedly higher than control ROs at 90 and 120 days (Figure 5A). Additionally, polymerase chain reaction (PCR) analysis revealed a significant elevation in the expression of FOS and EGR1, in both patient ROs compared to control ROs (Figure 5B,C).

### 2.5. Differential Expression of mRNAs between Control ROs and XLRS ROs at Day 90

**Figure 6:**
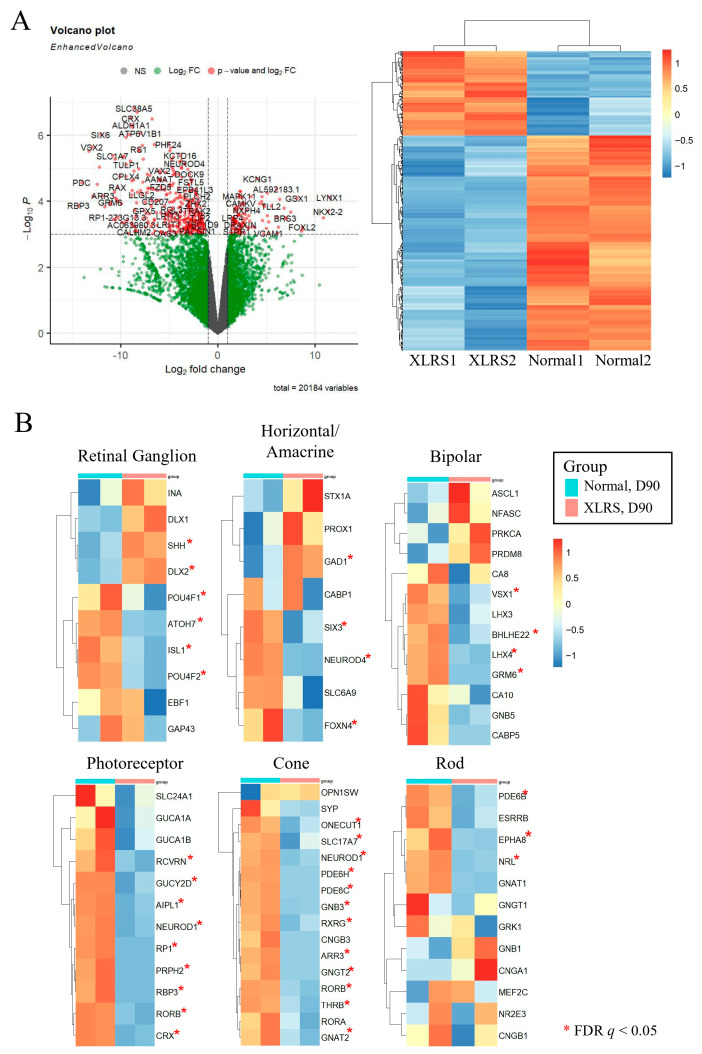
(**A**) Volcano plot and heatmap of differentially expressed genes comparing X-linked juvenile retinoschisis (XLRS) retinal organoids (ROs) with normal ROs at day 90. (**B**) Expression levels of retinal cell markers between XLRS ROs and normal ROs. * False discovery rate *q* < 0.05.

In DEG analysis for Control and XLRS ROs cultured for 90 days, 151 genes exhibited upregulation (fold change > 2, FDR q < 0.05) and 386 genes downregulation (fold change < 0.5, FDR q < 0.05) in the XLRS ROs compared to the control ROs (Figure 6A). XLRS ROs showed lower expression of retinal cell markers than those of Normal-ROs, especially for cone photoreceptor cells (Figure 6B).

**Figure 7:**
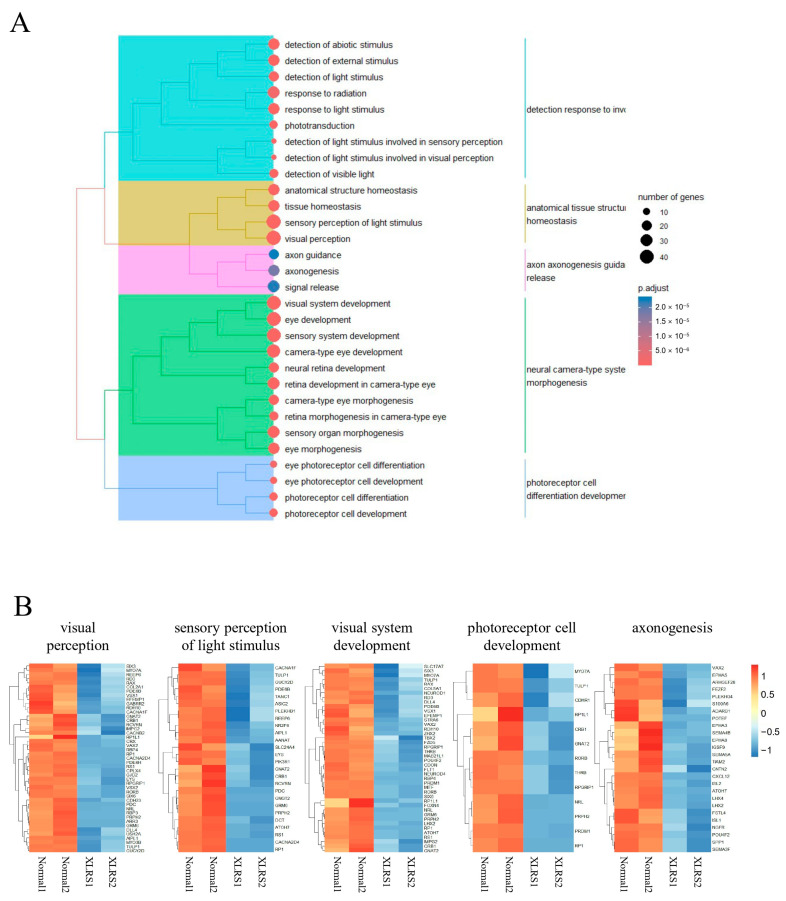
(**A**) Clustering analysis of the top 30 gene ontologies (biological processes) enriched by 386 downregulated genes in X-linked juvenile retinoschisis retinal organoids (ROs) compared to normal ROs at day 90. The right side displays the hierarchies of ontologies, while the left side presents key terms for each cluster. (**B**) Expression levels of genes within the most significantly enriched terms of ontologies of each cluster.

The 386 downregulated genes at day 90 were associated with biological processes such as visual perception, visual system development, and eye development (Figure 7A). The top 30 ontology terms could be clustered into 5 clusters; Cluster 1 included terms such as “detection”, “response to”, and “light”; Cluster 2 featured terms such as “anatomical”, “tissue”, “structure”, and “homeostasis”; Cluster 3 encompassed the terms “axon”, “axonogenesis”, “guidance”, and “release”; Cluster 4 contained terms such as “neural”, “camera-type”, “system”, and “morphogenesis”; Cluster 5 featured the terms “photoreceptor”, “cell”, “differentiation”, and “development” (Figure 7A). The expression level of genes in a representative ontology of each cluster were presented in Figure 7B.

### 2.6. Differential Expression of mRNAs between Control ROs and XLRS ROs at Day 120

**Figure 8:**
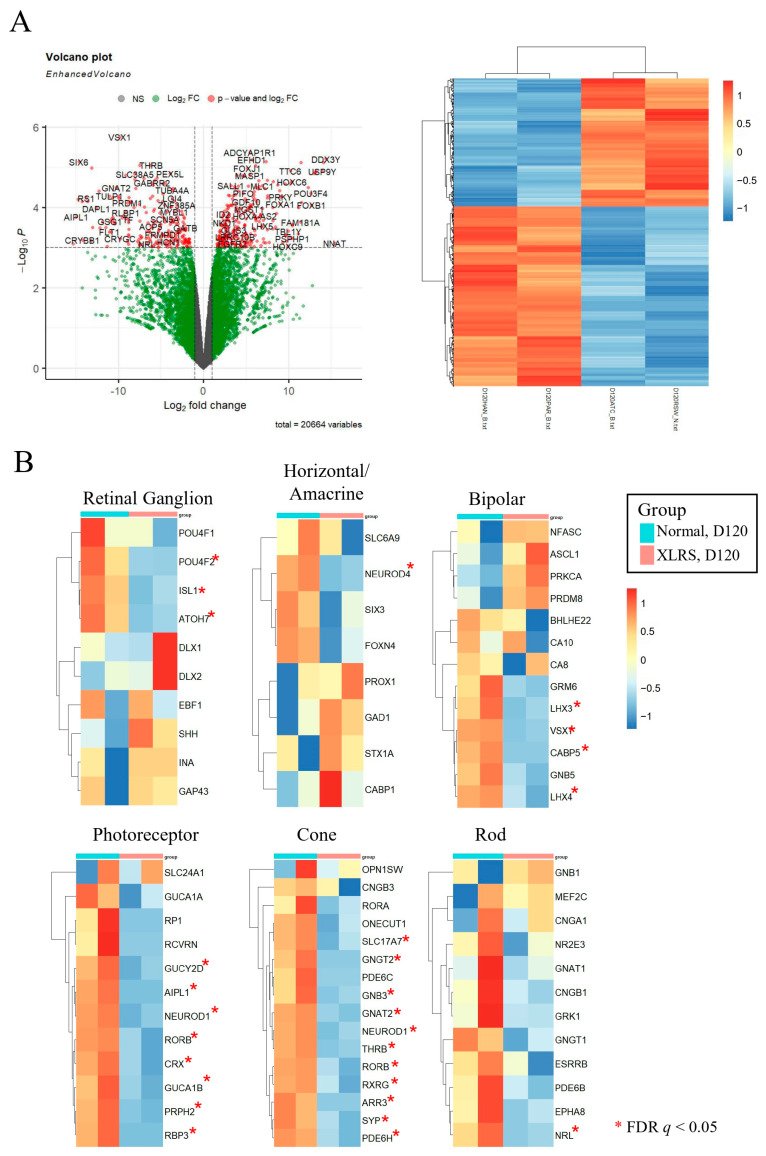
(**A**) Volcano plot and heatmap of differentially expressed genes comparing X-linked juvenile retinoschisis (XLRS) retinal organoids (ROs) with normal ROs at day 120. (**B**) Expression levels of retinal cell markers between XLRS ROs and normal ROs. * False discovery rate *q* < 0.05.

On day 120, 337 genes were upregulated and 241 were downregulated in the XLRS-ROs compared to the normal-ROs (Figure 8A). Similar to the results at day 90, XLRS-ROs showed lower expression of retinal cell markers than those of Normal-ROs, especially for cone and rod photoreceptor cells (Figure 8B).

**Figure 9:**
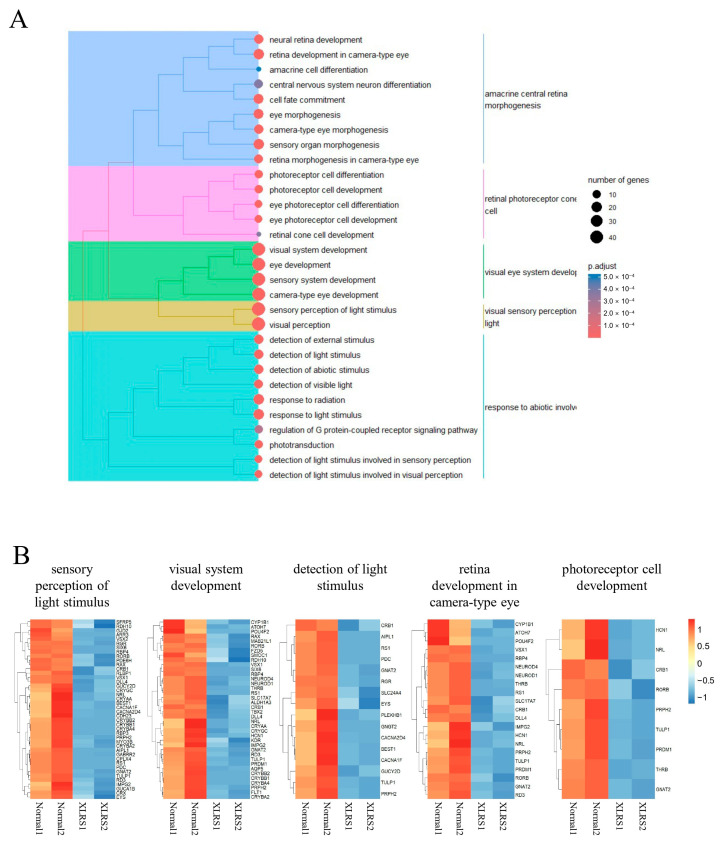
(**A**) Clustering analysis of the top 30 gene ontologies (biological processes) enriched by 241 downregulated genes in X-linked juvenile retinoschisis retinal organoids (ROs) compared to normal ROs at day 120. The right side displays the hierarchies of ontologies, while the left side presents key terms for each cluster. (**B**) Expression levels of genes within the most significantly enriched terms of ontologies of each cluster.

Gene ontology analysis showed that 241 downregulated genes in ROs cultured for 120 days were associated with visual system development, sensory perception of light stimulus, and photoreceptor cell differentiation (Figure 9A). The top 30 ontology terms could be clustered into 5 clusters; Cluster 1 included terms such as “amacrine”, “central”, “retina” and “morphogenesis”; Cluster 2 featured terms such as “retinal”, “photoreceptor”, “cone”, and “cell”; Cluster 3 encompassed the terms “visual”, “eye”, “system”, and “development”; Cluster 4 contained terms such as “visual”, “sensory”, “perception”, and “light”; Cluster 5 featured the terms “response to”, “abiotic”, and “involved” (Figure 9A). The expression level of genes in a representative ontology of each cluster were presented in Figure 9B.

### 2.7. Co-Culture of Control ROs Restores Pathological Phenotype of XLRS Patient ROs

**Figure 10:**
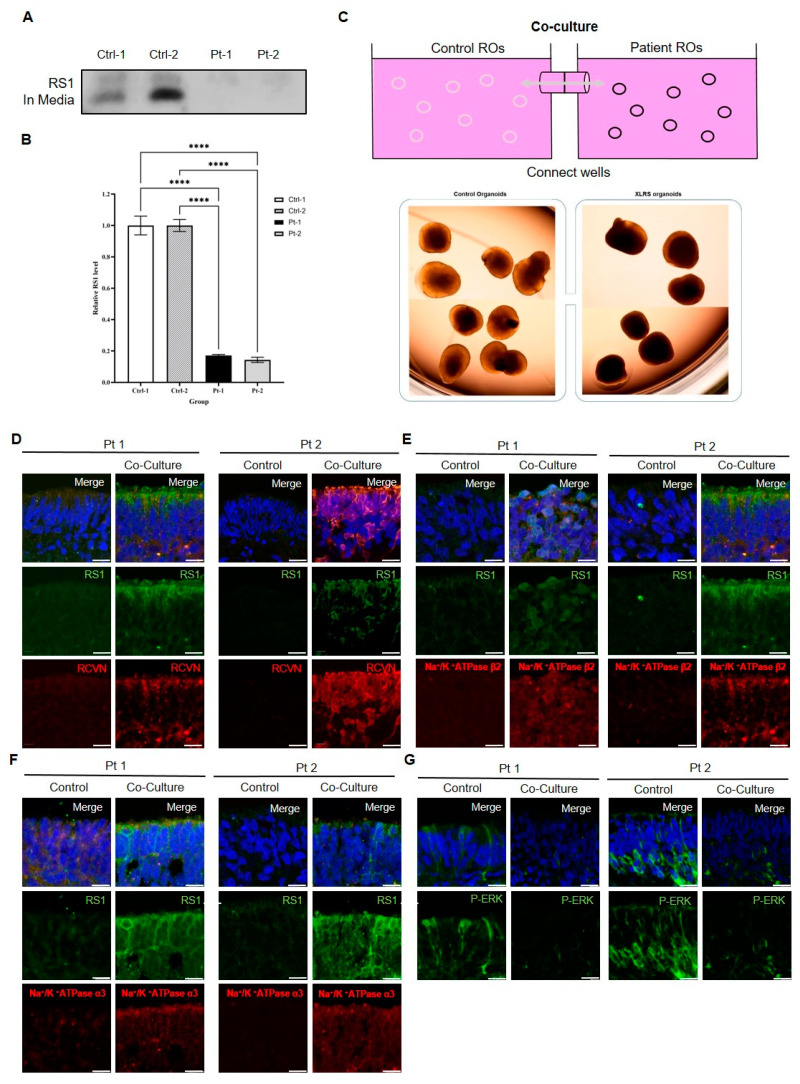
Effect of co-culture of control and X-linked juvenile retinoschisis patient retinal organoids (ROs). (**A**) Western blot analysis of the expression of the retinoschisin (RS1) protein in the culture medium of control ROs at days 90. (**B**) Quantitative analysis of band density. Each experiment is executed in triplicate. Data are shown as mean ± standard error (*n* = 3; **** *p* < 0.001, one-way analysis of variance). (**C**) Co-culture experiment involving control ROs and patient ROs using co-culture plate. (Gray circle: Control ROs, Black circle: Patient ROs, Arrow: Medium flow direction) (**D**) Immunofluorescence staining of RS1 (green) and photoreceptor marker recoverin (RCVRN) (red) in patient ROs following co-culture (scale bar = 50 µm). (**E**) Immunofluorescence staining of RS1 (green) and retinal Na/K-ATPase subunits α3 (ATP1A3) (red) in patient ROs following co-culture (scale bar = 50 µm). (**F**) Immunofluorescence staining of RS1 (green) and retinal Na/K-ATPase subunits β2 (ATP1B2) (red) in patient ROs following co-culture (scale bar = 50 µm). (**G**) Immunofluorescence staining of p-44/42 (green) in patient ROs following co-culture (scale bar = 50 µm).

Following our investigation into retinoschisin secretion during the differentiation process of control ROs, media collected from control ROs cultured for 90 days revealed the presence of RS1 protein, as confirmed by Western blot analysis. In contrast, few retinoschisin was detected in media from patient ROs (Figure 10A,B). Based on these findings, we assessed the effect of retinoschisin secreted by control ROs on XLRS patient ROs. To address this, we performed a co-culture experiment involving control ROs and patient ROs for 30 days, spanning from day 90 to day 120 of differentiation (Figure 10C). We aimed to discern any phenotypic changes in the patient ROs subsequent to the co-culture period.

Immunostaining of 120-day co-cultured patient ROs revealed a significant increase in the expression of RS1 and RCVRN, indicative of augmentation in the number of photoreceptor cells (Figure 10D). Additionally, the expression levels of ATP1A3 and ATP1B2 exhibited marked elevation following co-culture (Figure 10E,F). Conversely, the expression of Erk signaling showed a significant decrease post-co-culture (Figure 10G). These findings suggest that the co-culture of control ROs restore the pathological phenotype of XLRS patient ROs.

We then performed co-culture experiments involving isogenic control ROs and RS1-KO ROs for 30 days. The expression of RS1 and RCVRN did not significantly improve following co-culture. Additionally, the expression of ATP1A3, ATP1B2, and Erk signaling also showed no significant change after co-culture (Supplementary Figure S1).

## 3. Discussion

XLRS is a bilateral, hereditary, progressive retinal degenerative disorder likely manifesting at birth. The *RS1* gene encodes the retinoschisin critical for XLRS, with numerous human RS1 mutations leading to dysfunctional protein and disrupting retinal layer integrity [29]. While RS1-KO mouse or rat models have aided in understanding XLRS mechanisms and drug testing, significant gaps persist in gene variability and structural understanding [30,31,32]. Advancements in 3D culture of stem cell-derived ROs now allow the generation of well-structured retina-like tissue with mature, physiologically relevant photoreceptors [22,33,34]. Previous studies have attempted to establish 3D retinal organoids from the patient-specific hiPSCs for disease modeling of XLRS. Additionally, Huang et al. demonstrated CRISPR/Cas9 correction of *RS1* mutation rescued XLRS pathological phenotypes, while Duan et al. showed improvement in photoreceptor development delay by adeno-associated virus (AAV)-mediated gene augmentation with RS1 [35,36]. In our study, ROs derived from XLRS-hiPSCs were employed to establish a human in vitro model of XLRS. We also developed RS1-KO ROs by creating RS1-KO iPSCs through CRISPR/Cas9 gene editing. Using these 3D-RO model of XLRS, we successfully characterized the early features of XLRS diseases during RO development.

In our in vitro modeling of human retinal development, we have demonstrated that XLRS ROs exhibit a defect in retinoschisin production and a reduction in the expression of photoreceptor cells compared to control ROs. These results are consistent with previous results that RS1 KO mouse or rat exhibited progressive photoreceptor degeneration [37,38]. Notably, XLRS ROs showed that the retinal Na/K-ATPase subunit isoforms ATP1A3 and ATP1B2, as direct interaction partners of RS1, were significantly decreased, aligning with previous studies [27,39]. These Na/K-ATPases were known to play a crucial role in regulating intracellular signaling cascades, including the MAP kinase pathway [40,41,42]. Our study has also demonstrated a significant increase in Erk signaling during the early development of XLRS ROs compared to control ROs. It would thus be conceivable that disturbances of Erk signaling by RS1 deficiency might be one of the initial steps in XLRS pathology. These results suggest that disruption of the RS1-Na/K-ATPase signalosome complex due to RS1 deficiency may lead to defective MAP kinase regulation by the Na/K-ATPase.

In transcriptome analysis, XLRS ROs showed lower expression of retinal markers especially associated with photoreceptors than normal ROs at both 90 and 120 days. The downregulated genes in XLRS ROs were associated with visual perception, visual system development and photoreceptor cell development. These observations strongly suggested that the lack of retinoschisin could cause the impairment of photoreceptor development and subsequent loss of visual function. In protein–protein interaction network analysis, the key genes of downregulated genes in XLRS ROs compared to normal ROs was CRX, PRPH2, GUCY2D, AIPL1, and NRL, which has been known to be associated with various retina diseases. Pan et al. [43], recently revealed that CRX haploinsufficiency caused dysregulation of photoreceptor precursor translocation and differentiation using ROs. Mutations in the PRPH2 gene have been known to cause a wide range of autosomal dominant retinal dystrophies [44]. Mutations in GUCY2D, a crucial gene in the phototransduction process of cones and rods, is the most common causes of autosomal-dominant cone–/cone–rod dystrophies [45]. Missense and nonsense variants in the FKBP-like and tetratricopeptide repeat domains of AIPL1 cause Leber congenital amaurosis due to both the absence of HSP90 interaction and the impairment of PDE6 activity [46]. NRL is a factor important for rod photoreceptor cell differentiation and homeostasis, which is regarded as a therapeutic target to treat retinitis pigmentosa [47]. These pieces of evidence suggest that abnormality of retinoschisin could be linked to photoreceptor development via interaction with various disease-related genes and pathways. This possibility needs to be revealed through further research.

During the development of ROs, retinoschisin, a multisubunit protein, is expressed and secreted from both photoreceptor cells in the outer retina and bipolar cells in the inner retina [11,48]. We anticipated retinoschisin secretion during RO development and measured its presence in the culture medium. We observed an increase in retinoschisin in the culture medium starting around 90 days, coinciding with the initiation of photoreceptor development within the ROs. To investigate the effect of secreted retinoschisin on XLRS patient ROs, we performed co-culture experiments of control and patient ROs for 30 days. Co-culture systems are utilized for culturing and differentiating cells in vitro and are known to be greatly important in drug development processes as well as the treatment of incurable pathologies. Following co-culture, we found a significant increase in retinoschisin expression within patient ROs. Additionally, the expression of ATP1A3 and ATP1B2 was markedly elevated, while Erk signaling decreased. These findings suggest a therapeutic effect of control RO derived retinoschisin on photoreceptor cells of patient ROs.

However, unlike patient ROs, RS1-KO ROs did not exhibit photoreceptor recovery following co-culture. This discrepancy between patient ROs and RS1-KO ROs suggests that limited effects of co-culture are likely to be seen due to more pronounced photoreceptor development impairment in RS1-KO ROs. Both Pt-1 and Pt-2 carry mutations (Pro192Ser and Gln121ArgfsTer5) in the discoidin (DS) domain. A recently developed tool AlphaFold, a neural-network-based model for unknown protein structure prediction [49], showed that the mutation in Pt-1 results in relatively normal protein synthesis, folding, and disulfide-linked dimerization, but it is known to fail to further oligomerize into an octameric complex because this residue is located on subunit interface [50]. On the other hand, the mutation in Pt-2 enables the nascent polypeptide chain to be transported into the ER lumen, but the protein fails to fold into a native conformation due to the lack of two intramolecular disulfide bonds (C63–C219 and C110–C142) stabilizing the structure of DS domain and one intermolecular disulfide bridge involved in octamer formation [9]. In both mutations, the retinoschisin is abnormal, but it retains an intact RS1 domain and some DS domains in its structure, suggesting that this may have contributed to photoreceptor cell development. In contrast, RS1-KO ROs carry a frameshift deletion mutation in Exon 2 of the RS1 gene, resulting in no retinoschisin production and complete impairment of photoreceptor development. Even in the case of gene therapy, it is similar to what is known that the effect is limited when photoreceptor cells are significantly damaged. In other words, the limited effect of retinoschisin means that its effect may be restricted when photoreceptor cell development is severely impaired.

In present study, we performed co-culture experiments during the early stages of RO differentiation, when photoreceptor cells begin to develop, and demonstrated improvement of the pathological phenotype in XLRS ROs. However, we did not confirm whether co-culture is effective at the late stage of RO differentiation when the photoreceptor maturation occurs. Furthermore, although retinoschisin plays a crucial role in photoreceptor development, it may be difficult to attribute the recovery of photoreceptors solely to the effect of retinoschisin after co-culture. It cannot be ruled out the possibility that a variety of proteins, exosomal miRNAs, and other factors secreted from ROs might have influenced the development of photoreceptors in XLRS ROs. Further in-depth studies are needed in the future to determine whether RS1 and other factors can be utilized for treatment at the appropriate time, or if treatment options will indeed be available for patients with XLRS.

In conclusion, we demonstrated that *RS1* mutations result in defects in retinoschisin production and photoreceptor cell development in the XLRS RO model. Transcriptomic analyses of XLRS ROs also revealed decreased expression of retinal cells, especially photoreceptor cells. XLRS ROs exhibited dysregulation of Na/K-ATPase due to RS1 deficiency and increased Erk signaling pathway. Furthermore, partial recovery of the XLRS phenotype occurred when co-cultured with control ROs during the early stages of differentiation. Our data revealed, for the first time, the therapeutic effect of secreted RS1 protein on photoreceptor development in XLRS patient ROs. Collectively, our in vitro model system can be useful for investigating the pathophysiological mechanisms of XLRS diseases and optimizing treatment approaches.

## 4. Materials and Methods

### 4.1. Ethic Statements and Human Samples

The experimental procedures and protocols involving human samples were conducted according to the tenets of the Declaration of Helsinki and were approved by the Institutional Review Board of Soonchunhyang University Bucheon Hospital (Project identification # SCHBC 2020-08-024; approval date 15 October 2020). Human blood samples were obtained after taking informed consent from the patients.

### 4.2. Generation of iPSC from PBMCs

Human iPSCs derived from PBMCs were generated based on a previous report [51]. Briefly, PBMCs were isolated by density gradient centrifugation with Ficoll-Paque Plus (GE Healthcare, Uppsala, Sweden). PBMCs were cultured for 7 days in StemSpanTM SFEMII with StemSpan erythroid expansion supplement (Stem Cell Technologies, Vancouver, B.C., Canada). PBMCs were electroporated with episomal iPSC reprogramming plasmids (Addgene, Watertown, MA, USA), pCXLE-hOCT3/4-shp53, pCXLE-hSK, pCXLE-hUL, and pCXLE-EBNA1, using the 4D-Nucleofector X Unit (Lonza, Basel, Switzerland) with a P3 Primary Cell 4D-Nucleofector X kit (Lonza). Transduced cells were seeded on culture dishes coated with iMATRIX-511 (Takara, Tokyo, Japan) and cultured with StemFit Basic02 media (Ajinomoto, Tokyo, Japan). Once iPSC-like colonies formed, the media were switched to with E8 medium (Invitrogen, Carlsbad, CA, USA) during the course of 4 days. The colonies were picked, plated on Vitronectin (Invitrogen)-coated 12-well plate, and cultured with E8 medium containing ROCK inhibitor Y-27632.

### 4.3. Generation of RS1 KO iPSC by CRISPR/Cas9

The RS1-KO iPSCs were produced and provided by Dr. Junwon Lee. The RS1 gRNA 5′ GGTAGACGATAATCCCAATG-3′, targeting exon 2 of the RS1 gene, was cloned into the plasmid encoding the sgRNA (pRG2; #104174, Addgene, Watertown, MA, USA) using the BsaI restriction enzyme (New England Biolabs, Ipswich, MA, USA) and T4 DNA ligase (Takara, Shiga, Japan). Normal human male iPSCs were transfected with plasmid encoding SpCas9 (pRGEN-Cas9-CMV/T7-Puro-RFP; ToolGen, Seoul, Republic of Korea) and pRG2-RS1 gRNA using the Neon electroporation system (Thermo Fisher Scientific, Waltham, MA, USA) at 1300 voltages, 30 milliseconds, and 1 pulse. Puromycin was used for selection of transfected cells, and iPSCs clones were established by following single-cell dissociation and clonal expansion. The KO of RS1 was confirmed by sequencing of the target region.

### 4.4. hiPSC Cultures

The hiPSCs were maintained in Essential 8 (E8) medium (GibcoTM; Thermo Fisher Scientific) on vitronectin-coated culture dishes (Thermo Fisher Scientific). The cells were cultured at 37 °C in a standard incubator with 5% CO_2_ and 95% air, with daily medium changes. When cells reached approximately 70% to 80% confluency, they were mechanically passaged using the enzyme-free reagent ReLeSR (Stem Cell Technologies) every 5 to 7 days. The detached cell aggregates were then collected in E8 medium and pipetted up and down to achieve a uniform suspension, which was subsequently reseeded at a ratio of 1/10 to 1/60, depending on confluence

### 4.5. Differentiation into 3D ROs

The ROs were differentiated from hiPSCs following the retinal differentiation protocol outlined by Lee et al [52]. The hiPSCs were maintained in E8 medium (Thermo Fisher Scientific) on vitronectin-coated culture dishes and dissociated using ReLeSR (Stem Cell Technologies). The dissociated cells were then plated on a low-attachment 6-well plate with E8 medium containing 3 µM ROCK inhibitor Y27632 (Tocris Biosciences, Abingdon, UK) and 3 µM Blebbistatin (Tocris Biosciences) on day 0 to induce embryoid body (EB) formation. As differentiation progressed, the EBs were gradually transitioned to neural induction medium (NIM) composed of Dulbecco’s Modified Eagle Medium/Nutrient Mixture F-12 (DMEM/F12; 1:1; Thermo Fisher Scientific), 1% N-2 supplement (Thermo Fisher Scientific), non-essential amino acids (NEAAs), and 2 µg/mL heparin (StemCell Technologies), replacing the E8 medium without ROCK inhibitor Y27632 and Blebbistatin (Tocris Biosciences). Day 0 was marked as the day of detachment, with medium changes occurring on days 1 (25% NIM), 2 (50% NIM), and 3 (100% NIM). On day 7, the EBs were transferred to 35-mm Matrigel-coated dishes (Corning Life Sciences, Tewksbury, MA, USA) containing NIM at a density of 150 EBs per dish. By day 15, the medium was switched to retinal differentiation medium (RDM), which included DMEM/F12 (3:1), 2% B-27 supplement without vitamin A (Thermo Fisher Scientific), NEAAs, and an antibiotic–antimycotic solution (Thermo Fisher Scientific), with medium changes occurring every other day. Between days 25 and 28, the loosely adherent central portions of the neural clusters were lifted using a P1000 pipette under an Evos XL cell imaging microscope (Invitrogen). The selected optic vesicle-like aggregates were then further cultured in RDM, supplemented with 10% exosome-depleted fetal bovine serum (Cat. No. A2720801; Thermo Fisher Scientific), 100 mM taurine (Sigma-Aldrich, St. Louis, MO, USA), and 2 mM L-alanyl-L-glutamine dipeptide (GlutaMAXTM; Thermo Fisher Scientific), to develop 3D ROs. For long-term culture, the medium was changed every 3 days until the desired developmental stage was reached

### 4.6. Immunocytochemistry

The ROs were fixed in 4% paraformaldehyde at room temperature for 30 min, then cryoprotected by immersion in 15% sucrose for at least 2 h, followed by 30% sucrose overnight. Subsequently, the samples were embedded in optimum cutting temperature (OCT) compound. The OCT blocks were sectioned into 10 μm thick slices and allowed to incubate at room temperature for at least 1 h before either proceeding with immunostaining or storing at −80 °C. For immunostaining, slides were first rinsed in 0.1% Triton X-100 (Sigma-Aldrich) in PBS and then blocked with 5% donkey serum in PBS for 1 h. Primary antibodies were diluted in the blocking solution and incubated overnight at 4 °C. The primary antibodies used were: RS1 (1:1000; Abcam, Cambridge, UK), RCVRN (1:100; Millipore, Burlington, MA), Na/K-ATPase α3 (1:500; Almone Labs, Jerusalem, Israel), Na/K-ATPase β2 (1:500; Novus Biologicals, Centennial, CO, USA), phospho-p44/42 MAPK (phosphor-Erk1/2) (1:500; Cell Signaling Technology, Danvers, MA, USA), CRX (1:1000; Abnova, Taipei, Taiwan), and NRL (1:1000; R&D, Minneapolis, MN, USA). Following primary antibody incubation, species-specific secondary antibodies conjugated with Alexa Fluor 488 or 568 were diluted in antibody dilution buffer (1XPBS) and applied to the slides for 2 h at room temperature. Nuclear staining was performed using Hoechst^®^ 33,342 (Invitrogen). Fluorescence images were captured with a confocal microscope (DMI8; Leica Camera, Wetzlar, Germany).

### 4.7. Real-Time PC

The total RNA was extracted from ROs using TRIzol reagent (Invitrogen). First-strand cDNA was then synthesized with the SuperScript™ III First-Strand Synthesis System (Invitrogen) following the manufacturer’s instructions. For amplification, the cDNA was processed using the QuantiSpeed SYBR Hi-ROX Kit (PhileKorea, Daejeon, Republic of Korea) on a StepOnePlus Real-Time PCR System (Applied Biosystems, Carlsbad, CA, USA). The thermocycling conditions were set as follows: polymerase activation at 95 °C for 2 min, followed by 40 cycles of denaturation at 95 °C for 5 s, and annealing/extension at 60 °C for 30 s. Melt curve analysis was performed with the following steps: 95 °C for 15 s, 60 °C for 1 min, and a final 95 °C for 15 s. Glyceraldehyde 3-phosphate dehydrogenase (GAPDH) served as the internal control. mRNA expression levels were quantified using the 2^−ΔΔCT^ method. Primer sequences are provided in Table 1.

### 4.8. Western Blot Analysis

The RO media were concentrated using Amicon^®^ Ultra 2 mL Centrifugal Filters (UFC200324; Merck Millipore, Burlington, MA, USA) according to the manufacturer’s instructions. Protein samples were then mixed with an equal volume of 5× sodium dodecyl sulfate (SDS) sample buffer, boiled for 10 min at 98 °C, and separated using 12% SDS-polyacrylamide gel electrophoresis (SDS-PAGE). After electrophoresis, the proteins were transferred to polyvinylidene fluoride (PVDF) membranes. The membranes were blocked in 5% bovine serum albumin (BSA) for 1 h. Specific antibodies were applied: RS1 at a 1:1000 dilution (Abcam, ab167579) and β-actin monoclonal antibody at a 1:10,000 dilution (Sigma-Aldrich). Following primary antibody incubation, the membranes were washed three times with PBST, and then incubated for 2 h with horseradish peroxidase-conjugated anti-mouse or anti-rabbit IgG at a 1:5000 dilution (GenDEPOT, Baker, TX, USA). After a final set of three washes with PBST, the immuno-positive bands were visualized using a chemiluminescence reagent (ATTO Corp., Tokyo, Japan) and an Azure imaging system (Azure Biosystems, Dublin, CA, USA). Band intensity was quantified using ImageJ software version 1.53e (National Institutes of Health, Bethesda, MD, USA)

### 4.9. RNA Sequencing and Gene Ontology-Pathway Enrichment Analysis

Total RNA was isolated using Trizol reagent (Invitrogen). RNA quality was assessed by Agilent 2100 bioanalyzer (Agilent Technologies, Amstelveen, The Netherlands), and RNA quantification was performed using ND-2000 Spectrophotometer (Thermo Fisher Scientific). Libraries were prepared from total RNA using the NEBNext Ultra II Directional RNA-Seq Kit (New England Biolabs). The isolation of mRNA was performed using the Poly(A) RNA Selection Kit (LEXOGEN, Inc., Vienna, Austria). The isolated mRNAs were used for the cDNA synthesis and shearing, following manufacture’s instruction. Indexing was performed using the Illumina indexes 1–12. The enrichment step was performed using PCR. Following this, the libraries were assessed with the TapeStation HS D1000 Screen Tape (Agilent Technologies) to determine the mean fragment size. Library quantification was then conducted using a quantification kit with a StepOne Real-Time PCR System (Life Technologies, Carlsbad, CA, USA). High-throughput sequencing was performed as paired-end 100 sequencing using NovaSeq 6000 (Illumina, San Diego, CA, USA).

Quality control of raw sequencing data was performed using FastQC (Simon Andrews; Babraham Institute, Cambridge, UK; https://www.bioinformatics.babraham.ac.uk/projects/fastqc/ (accessed on 16 March 2022). Adapter and low-quality reads (<Q20) were removed using FASTX_Trimmer (Hannon Lab, Cold Spring Harbor Laboratory, New York, NY, USA; FASTX toolkit; http://hannonlab.cshl.edu/fastx_toolkit/ (accessed on 16 March 2022) and BBMap (Bushnell B; SourceForge Headquarters, San Diego, CA, USA; https://sourceforge.net/projects/bbmap/ (accessed on 16 March 2022). Then the trimmed reads were mapped to the reference genome using TopHat [53]. The read count was extracted using HTseq [54], and differential expression gene (DEG) analysis was performed by a generalized linear model quasi-likelihood F-test using EdgeR within R (R development Core Team; The R Foundation for Statistical Computing, Vienna, Austria) [55].

EnhancedVolcano (Bioconductor, Seattle, WA, USA; https://bioconductor.org/packages/EnhancedVolcano (accessed on 24 April 2024) and pheatmap in R (The R Foundation for Statistical Computing; https://CRAN.R-project.org/package=pheatmap (accessed on 24 April 2024) packages were used to visualize the results of DEG analysis. Gene ontology and KEGG pathway enrichment analyses were performed using cluterProfiler package [56] with default options. The similarity of obtained gene ontology or KEGG pathway were calculated and visualized using enrichplot package (Bioconductor; https://bioconductor.org/packages/enrichplot (accessed on 24 April 2024).

### 4.10. Co-Culture Techniques

The co-culture experiment was performed using the UniWells™ Horizontal Co-Culture Plate (Catalog No. 384-14421; FUJIFILM Wako Chemicals, Richmond, VA, USA), as recently described by Shimasaki et al [57]. Equal numbers of control ROs and XLRS ROs, between 5 and 10, were cocultured for 30 days, from 90 to 120 days of differentiation. The media were replaced every 3 days. During these media changes, observations were made to monitor any visible changes or effects resulting from the co-culture conditions. Co-cultured XLRS ROs were used to investigate pathological phenotypic changes, such as those in RS1, retinal Na/K-ATPase, and the Erk signaling pathway.

### 4.11. Statistical Analysis

Each experiment was independently repeated at least three times. The differences were statistically analyzed using a one-way analysis of variance (ANOVA) with GraphPad Prism software version 10.2.3 (GraphPad Software, Inc., San Diego, CA, USA). Significant differences are denoted by single (#; *p* < 0.05), double (##; *p* < 0.01), or triple asterisks (###; *p* < 0.001).

## Figures and Tables

**Table 1:** List of primers used for quantitative real-time polymerase chain reaction.

Gene	Forward	Reverse
c-FOS	ACTAACCACTCACCCGCAGAC	CCAGGTCCGTGCAGAAGT
EGR1	AGCCCTACGAGCACCTGAC	GGTTTGGCTGGGGTAACTG

## Data Availability

Data will be made available on request.

## Supplementary Materials

The following supporting information can be downloaded at: https://www.mdpi.com/article/10.3390/ijms25158203/s1.
