# Supplementary material for: Early Developmental Characteristics and Features of a Three-Dimensional Retinal Organoid Model of X-Linked Juvenile Retinoschisis
Source: Int J Mol Sci. 2024 Jul 27;25(15):8203. doi: 10.3390/ijms25158203 (PMC11311801; doi:10.3390/ijms25158203)

Supplementary Figure S1. Effect of co-culture of control and RS1-KO retinal organoids (ROs). (A) Immunofluorescence staining of RS1 (green) and photoreceptor marker recoverin (RCVRN) (red) in RS1-KO ROs following co-culture. (B) Immunofluorescence staining of RS1 (green) and Na/K-ATPase subunits β2 (ATP1B2) (red) (red) in RS1-KO ROs following co-culture. (C) Immunofluorescence staining of p-44/42 (green) in RS1-KO ROs following co-culture. (scale bar = 50 μm).

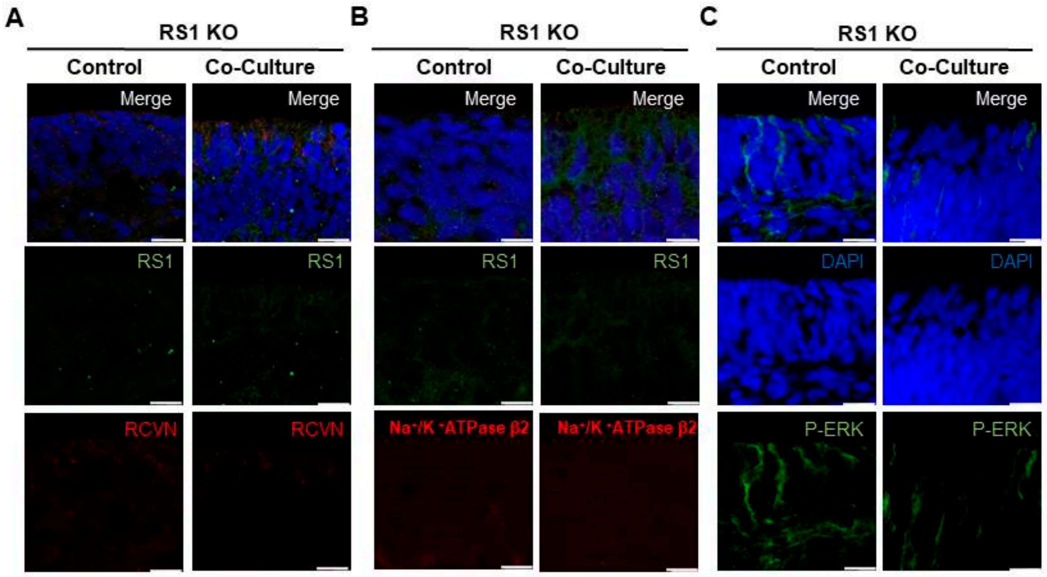

Supplement: Supplementary file 1 [file ijms-25-08203-s001.zip › Supplementary Figure S1.pdf]
