# Supplementary material for: Early Developmental Characteristics and Features of a Three-Dimensional Retinal Organoid Model of X-Linked Juvenile Retinoschisis
Source: Int J Mol Sci. 2024 Jul 27;25(15):8203. doi: 10.3390/ijms25158203 (PMC11311801; doi:10.3390/ijms25158203)
Supplement: Supplementary file 1 [file ijms-25-08203-s001.zip › Supplementary Table S1.pdf]

Supplementary table

	Gender	AGE	RS1 Mutation	BCVA	Fundus examination	Optical Coherence Tomography
**Patient-1**	Male	18	c.574C>T (p.Pro192Ser)	OD) 0.6 OS) 0.6	Foveal schisis No peripheral retinoschisis	small cystic-like spaces peri-foveal and large cystic-like spaces within the fovea
**Patient-2**	Male	41	c.362delA (Gln121ArgfsTer5)	OD) 0.3 OS) 0.3	Foveal schisis No peripheral retinoschisis	small cystic-like spaces peri-foveal and large cystic-like spaces within the fovea
**Control-2**	Male	54	No mutation	OD) 1.0 OS) 1.0	Normal	Normal
